# Gut dysbiosis in stroke and its implications on Alzheimer’s disease‐like cognitive dysfunction

**DOI:** 10.1111/cns.13613

**Published:** 2021-01-19

**Authors:** Justin Cho, You Jeong Park, Bella Gonzales‐Portillo, Madeline Saft, Blaise Cozene, Nadia Sadanandan, Cesar V. Borlongan

**Affiliations:** ^1^ Department of Neurosurgery and Brain Repair University of South Florida Morsani College of Medicine Tampa FL USA; ^2^ Northwestern University Evanston IL USA; ^3^ University of Michigan Ann Arbor MI USA; ^4^ Tulane University New Orleans LA USA; ^5^ Georgetown University Washington DC USA

**Keywords:** Alzheimer's disease, chronic stroke, cognitive decline, gastrointestinal microbiome

## Abstract

Various neurological disorders, such as stroke and Alzheimer's disease (AD), involve neuroinflammatory responses. The advent of the gut‐brain axis enhances our understanding of neurological disease progression and secondary cell death. Gut microbiomes, especially those associated with inflammation, may reflect the dysbiosis of both the brain and the gut, opening the possibility to utilize inflammatory microbiomes as biomarkers and therapeutic targets. The gut‐brain axis may serve as a contributing factor to disease pathology and offer innovative approaches in cell‐based regenerative medicine for the treatment of neurological diseases. In reviewing the pathogenesis of stroke and AD, we also discuss the effects of gut microbiota on cognitive decline and brain pathology. Although the underlying mechanism of primary cell death from either disease is clearly distinct, both may be linked to gut‐microbial dysfunction as a consequential aberration that is unique to each disease. Targeting peripheral cell death pathways that exacerbate disease symptoms, such as those arising from the gut, coupled with conventional central therapeutic approach, may improve stroke and AD outcomes.

## Graphical abstract

Gut microbiota and stroke. A potential stroke therapy involves modification of the gut microbiota via prebiotics and probiotics, triggering the production of therapeutic metabolites (i.e., anti‐inflammatory factors) that may sequester the secondary cell death associated with stroke.

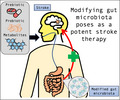

## 1 INTRODUCTION

Stroke is the fifth leading cause of death in the United States. Unfortunately, limited treatment options are available in both acute and chronic settings. Although primary cell death is caused by acute deprivation of oxygen and nutrients, secondary cell death caused by inflammation plays an integral role in the disabling neurological deficits often observed in stroke patients. Therefore, mediating the post‐stroke inflammatory response and preventing secondary cell death may be a valuable therapeutic target.

The mechanism underlying stroke progression was initially thought to be isolated to the central nervous system (CNS). However, increasing evidence points to the critical role of the peripheral nervous system in secondary cell death post‐stroke. Specifically, the gut‐brain axis may be involved in stroke progression as evidenced by the abnormal increase of inflammation observed in the brain and the gut following stroke.^1^, ^2^, ^3^ Normal gut functions and structures are maintained by microorganisms and bacteria, including pro‐inflammatory microbiomes. Other peripheral microbiomes have been characterized in the skin, oral cavity, vagina, and even the brain.^4^ Gut dysbiosis caused by dysfunction of the immune system and altered metabolism may influence the interaction between the gut and brain during the onset of stroke.

While the enteric nervous system, or gastrointestinal (GI) tract, functions independently to the CNS, digestive activities involve parasympathetic and sympathetic control, which connects the CNS and the GI. Furthermore, neural fibers linking the brain and gut allows for both the relay of sensory information to the CNS and CNS regulation of GI function. Following an insult to the CNS‐like stroke, the gut‐brain axis normally involved in maintaining homeostasis is activated to regulate dysbiosis.^5^ Inflammatory activity in the gut may be reflected in the brain microbiome^6^ in the ischemic penumbra, highlighting a mirrored activity in both the brain and gut and suggesting a new approach to stroke pathology and treatment. However, further investigations must be conducted to confirm the existence of brain microbiome and understand its potent applications in the gut‐brain axis and neurological consequences.

This peripheral shift in treating cognitive disorders, specifically therapeutic strategies outside the CNS has been accepted by the field of stem cell‐based regenerative medicine. Transplantation of cells, microvesicles, microRNAs, and other molecules has encouraged the incorporation of peripheral therapeutic targets in organs other than the brain.^7^, ^8^, ^9^, ^10^ Characterizing gut and brain microbiomes, in addition to microbiomes located in other organs, using single‐cell omics and transcriptomics^11^, ^12^ will be the first step in elucidating the stroke pathology and progressing cell‐based therapeutic strategies along with other neurological diseases.^7^ Furthermore, Parkinson's disease (PD) models have recently revealed the use of potent microbiomes both as a biomarker and therapeutic target,^13^, ^14^ shifting from brain‐focused neurological diagnosis and treatments to analysis of peripheral in the progression of PD.^15^ In fact, abnormal GI symptoms appear before motor symptoms in PD, suggesting that gut dysbiosis occurs prior to the onset of brain pathology.^13^, ^14^ Therefore, investigating and targeting the peripheral source of PD, if any, may be more effective than the palliative therapies used today.

Parkinson's disease models have detected higher frequencies of α‐synuclein in PD patients compared to healthy patients. Studies have also highlighted neuronal inflammation triggered by bowel inflammation, inducing neuronal loss, and enhancing PD symptoms.^16^, ^17^ Beneficial anti‐inflammatory bacterial species were shown to be present at significantly lower concentrations while pro‐inflammatory bacteria, such as *Ralstonia*, were abundant in PD patients.^18^ Our two recent studies identified three distinct gut microbiotas, namely LAB158, BAC303, and EREC482, that were overexpressed after neurotoxin lesions and abrogated by stem cell treatment. The idea that pro‐inflammatory gut microbiomes and mechanisms may lead to neurodegeneration in PD^16^, ^18^, ^19^ introduces a possible reoccurrence of similar dysbiotic gut‐brain axis in secondary cell death observed in stroke.^20^ Therapeutic models have suggested that altering gut microbiome populations may improve PD outcomes. Consuming fermented milk for approximately 4 weeks was seen to improve PD symptoms, such as constipation.^21^ Although anti‐TNF and immunosuppressant treatments were shown to reduce PD risks,^22^, ^23^, ^24^ limited evidence has supported the use of probiotics for PD treatment. However, emerging evidence from recent studies has highlighted anti‐inflammatory and gut dysbiosis restoration mechanisms,^25^ proposing probiotic administration as a potential PD therapy.

Characterization of specific gut and brain microbiomes may uncover homeostasis and microenvironmental dysbiosis associated with a brain with normal, healthy functions and one that drives neurodegeneration. Single‐cell omics and stem cell therapy may serve as potent tools to examine the gut‐brain axis in stroke and neurological disorders. With aberrant protein aggregation seen in PD also accompanying Alzheimer's disease (AD), and with AD‐like cognitive impairment recognized in stroke, understanding the role of the gut‐brain axis in stroke and AD may reveal novel insights into the pathology and treatment of these diseases. Accordingly, this review focuses on the potential contribution of gut dysbiosis to stroke and AD with a focus on presenting microbiota and brain pathology that mediate the rampant cognitive decline in both diseases.

## 2 PATHOLOGY OF CHRONIC STROKE PRESENTING AS COGNITIVE IMPAIRMENTS

Approximately 25%–30% of stroke patients develop immediate or delayed cognitive impairment or vascular dementia after suffering an ischemic stroke.^26^ Cognitive impairment or dementia after stroke is defined as dementia that primarily occurs three months after stroke onset. Risk factors for developing cognitive impairment or dementia include older age, family history, genetic variants, vascular comorbidities, and prior ischemic stroke or recurrent illness.^26^ Additionally, medical conditions such as hypertension, diabetes, obesity, and dyslipidemia are associated with a higher risk of cognitive decline and dementia.^27^ To reduce the burden of cognitive dysfunction after stroke, it is imperative to control vascular disease risk factors and understand the mechanisms of dementia after stroke injury. The neuroanatomical lesions in specific areas, such as the hippocampus and the white matter lesions (WMLs), caused by stroke and cerebral microbleeds (CMBs) due to small cerebrovascular diseases contribute to the pathogenesis of post‐stroke cognitive impairment.^28^

Ischemic stroke‐induced dementia has been considered to be caused by the neuroanatomical lesions as previously described. A past study conducted by Tomlinson et al. supported the notion that infarcts in specific areas of the brain, such as the hippocampus and entorhinal cortex, serve as a key factor for the mechanisms of cognitive impairment and are associated with the severity of dementia.^29^ Recent studies have demonstrated that WMLs serve as the common demonstrations of damage in the cerebral parenchyma due to the small cerebrovascular disease.^28^ Post‐stroke survivors who exhibit white matter hyperintensities volumes are likely to experience shorter time to dementia onset and blood brain barrier (BBB) damage.^26^ Additionally, small vessel disease plays a prevalent role in stroke pathophysiology and is the leading cause of cognitive decline and functional loss, especially in older patients.^27^ Understanding stroke pathology in terms of cognitive behavior may introduce new therapeutic approaches for stroke patients.

To assess strategic regions of the brain for post‐stroke cognitive impairment, multivariate lesion‐mapping can be used on ischemic stroke patients. Strategic structures for cognitive impairment after stroke include the left angular gyrus, left basal ganglia structures, and the white matter around the left basal ganglia.^30^ However, further studies are needed to develop more comprehensive models for post‐stroke cognitive impairment and better understand the brain histology.

Patients who suffer from a stroke have cerebral compromise and cognitive dysfunction.^26^ This causes stroke survivors to be at an increased risk for cognitive impairment.^31^, ^32^ Secondary cell death caused by neuroinflammation and immunodepression promotes further detrimental effects on the cognitive functions and physiological structure of the brain.^33^, ^34^, ^35^, ^36^, ^37^ Pro‐inflammatory mechanisms may also contribute to the pathways leading to dementia.^38^ Specifically, cerebral atrophy causes microglia and astrocytes to exhibit a dampened cytokine response in stroke‐induced dementia patients.^39^, ^40^, ^41^

Neuroinflammation and unregulated immune response after primary stroke lesions promote secondary cell death, causing further physiological damage to the brain and exacerbating cognitive dysfunction. Recent studies suggest that post‐stroke inflammation and cognitive deficits may also be linked to GI microbiota via the bidirectional communication between the brain and gut. Studies observing the effect of colonizing germ‐free mice with microbiota obtained from stroke mice showed that the recipient mice, after also experiencing cortical stroke, demonstrated larger infarct volumes when compared to nonrecipient mice. Additionally, recipient mice expressed higher levels of Th1 and Th17,^42^ the inflammatory T‐cells that may be involved in the pathogenesis of stroke, thus demonstrating a possible relationship between stroke‐induced neuroinflammation and gut microbiota. Specifically, altered microbiota may worsen post‐stroke inflammation, thereby, exacerbating physiological and cognitive dysfunctions. Other studies demonstrated a therapeutic link between neuroinflammation and microbiota alterations. In particular, anti‐inflammatory neuroprotective activities were revealed when antibiotics, specifically amoxicillin/clavulanic acid, were administered before inducing stroke. Post‐stroke infarct volume was reduced by 60% in administered mice compared to control groups.^43^ Furthermore, regulatory T‐cells were increased and levels of cells inhibiting effector T‐cells IL‐17+ γδ T decreased,^43^ promoting mechanisms of immune and neuroinflammation modulation. Although the effects of gut microbiome dysbiosis on post‐stroke cognitive alterations remain not well defined, an approach that reestablishes normal microbiome and enhances anti‐inflammatory cytokines could be beneficial in preventing cognitive dysfunction caused by stroke or by other neurological diseases.

To further understand the gut‐brain axis, evidence of stroke impact on gut microbiota was also reviewed. 50% of stroke patients are diagnosed with GI complications,^44^ hinting to the bilateral communication between the gut and brain. Signaling pathways involved in the gut‐brain communication include the vagus nerves, damage associated molecular patterns (DAMPs), and cytokines of gut inflammation. After stroke‐induced lesions occur, DAMPS and cytokines are released from the brain, activating gut inflammation and immune cells.^20^ Diverse gut microbiomes are severely reduced after stroke,^45^, ^46^ preventing the gut from maintaining homeostasis due to lack of communication between intestinal immune cells.^47^ Fecal samples of stroke patients reveal a significant change in the diversity of microbial populations in addition to increased intestinal barrier damage, which causes alterations in intestinal inflammatory and immune activity.^42^, ^48^ Dysfunction of the intestinal immune system allows the activation of gut γδ T‐cells which migrate to the stroke injury and initiates pro‐inflammatory cytokines to guide myeloid cells to the damage site, further inducing neuroinflammatory activity.^49^ To summarize, the top‐to‐bottom (brain‐to‐gut) pathway involves neuronal DAMPs and cytokines to circulate and disrupt healthy gut functions. As a result, gut inflammation and immune response activate γδ T‐cells that migrate to the brain in a bottom‐to‐top (gut‐to‐brain) manner and exacerbate neuronal inflammation and cognitive dysfunctions. Therefore, we believe gut dysbiosis may be relevant in stroke pathology and may be a consequence of stroke that may further induce neuroinflammation and cognitive impairment.^45^, ^48^, ^50^, ^51^ Targeting gut microbiota to mitigate further damage and regulate immune activity may be a potent stroke treatment.^2^, ^52^

## 3 GUT MICROBIOTA AND ALZHEIMER’S DISEASE

**FIGURE 1:**
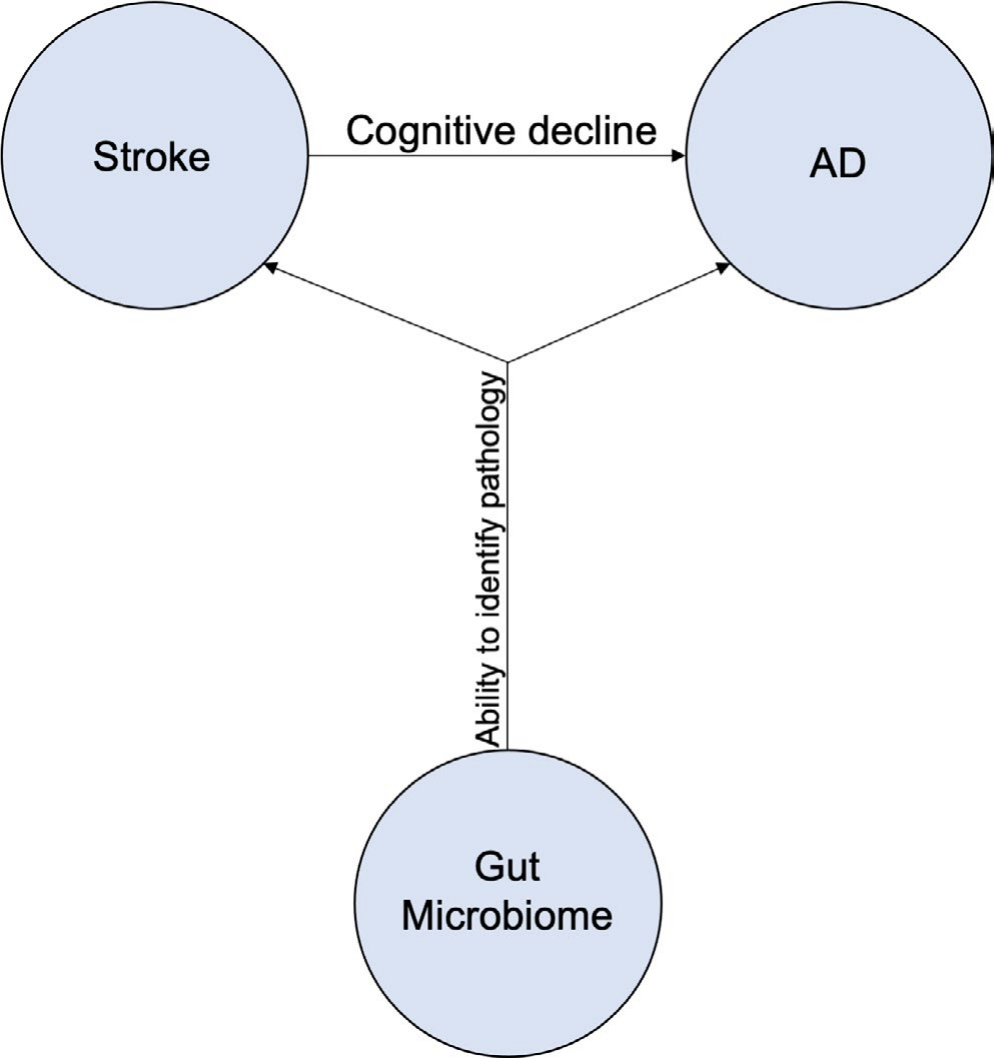
Stroke and Alzheimer's Disease possess similar cognitive behavior decline and brain pathology. Because gut microbiome is associated with cognitive impairments, such as AD‐induced dementia, stroke may also manifest with altered gut microbiota

Based on the dominant stroke‐induced dementia, investigations into Alzheimer's Disease, a neurodegenerative disease with a prevalent form of dementia that accounts for 50%–70% of all dementia cases,^53^ may provide a better understanding of this cognitive decline in stroke. Indeed, stroke demonstrates cognitive decline and brain pathology reminiscent of AD,^54^, ^55^, ^56^, ^57^, ^58^, ^59^, ^60^, ^61^, ^62^, ^63^ possibly due to the genetic similarities between AD and stroke.^64^ Consequently, stroke may also display alterations in the gut microbiome induced by AD‐like cognitive damage (Figure 1). Residing in the GI tract, the gut microbiome is composed of many highly influential microorganisms.^65^ Alteration of the gut microbiome population may exacerbate disease symptoms of gut disorders and CNS diseases, such as AD.^15^, ^25^ Fluctuation in microbiota influences the brain via the gut‐brain axis, a bidirectional system with immune, endocrine, neural, and metabolic functions.^65^, ^66^ Furthermore, dysbiotic microbiota populations may exacerbate BBB permeability, possibly mediating pathogenesis of AD and other CNS degenerative disorders.^67^ Bacterial secretion of amyloids and lipopolysaccharides may upregulate pro‐inflammatory cytokines through the gut‐brain axis or BBB.^65^ Elucidation of the underlying mechanisms is imperative to provide effective therapy that addresses multiple factors underlying the pathogenesis of AD and other neurodegenerative diseases.

Research utilizing animal AD models provide insight into how microbiome composition affects the brain. When fecal microbiomes and fecal short chain fatty acid composition (SCFAs) were compared between AD‐model mice and wild‐type, results indicated significant differences in gut microbe populations, SCFAs level fluctuation, and structural abnormalities in AD mouse intestine.^68^ The AD mice showed decreased levels of SCFAs which may consequently modify 30 metabolic pathways and further contribute to amyloid deposition, in turn, exacerbating the manifestation of AD.^68^ APP/PSI mice (AD mouse model) demonstrated a decline in spatial learning and memory, along with the presence of amyloid plaque and a distinct gut microbiome. As the APP/PSI mice aged, microbial diversity in the gut diminished. Compared to the wild‐type mice, APP/PSI mice displayed increased levels of Helicobacteraceae and Desulfovibrionaceae, as well as genuses Odoribacter and Helicobacter.^69^ Mutated transgenes were inserted into mice to further elucidate the effect of AD on the gut microbiota composition. 5xFAD mice, transgenic mice containing AD‐linked genes, were compared to wild‐type mice over three stages. 5xFAD mice demonstrated decreased body weight as the early stages of AD progressed. There was a significant decrease in trypsin concentration of 5xFAD mice compared to wild‐type, as well as alterations in microbiota as the mice aged. More importantly, genetic predisposal to AD may influence gut microbiota composition.^70^ Furthermore, when the gut‐brain axis was examined in an AD Drosophila model, the findings suggested that gut dysbiosis associated with AD spurs a significant reduction of Acetobacter and Lactobacilli, as well as a substantial decrease of acetate, stemming from the activity of SCFAs.^71^

The link between the gut microbiota and the AD‐afflicted brain seen in animal models can also be observed in patients. When compared to the control group, fecal samples from AD patients demonstrated significant variation in gut microbiome taxonomy with differing quantities of Bacteroids, Ruminococci, Actinobacteriae, Lachnospiraceae, and Selenomonadales.^72^ Another patient study revealed that fecal samples collected from AD‐induced dementia participants displayed significantly lower levels of diversity in gut microbiota compared to those without AD‐induced cognitive decline. The fecal samples from AD participants revealed a lower number of Firmicutes and Bifidobacterium and higher levels of Bacteroidetes, indicating a substantial change in gut microbiome.^73^ Moreover, the altered gut microbiota can be associated with AD‐biomarkers found in cerebrospinal fluid. Participants with mild cognitive impairment and those without cognitive decline underwent two diets: modified Mediterranean‐ketogenic diet (MMKD) and American Heart Association Diet (AHAD). In those afflicted with mild cognitive damage (MCI), the levels of Proteobacteria and cerebrospinal fluid AD‐biomarkers Aβ‐42 and Aβ‐40 displayed a positive correlation. On the other hand, propionate and butyrate demonstrated a negative correlation with those same biomarkers. The two diets had varied impacts on the gut microbiome among normal and cognitively injured participants. Both diets spurred an increase in phylum Tenericutes, especially in MCI subjects, which was associated with a decrease in Aß42 in cerebrospinal fluid. MMKD‐induced elevation of Enterboacterieae was correlated with a reduction of Aß42 in MCI participants.^74^

Due to overwhelming evidence highlighting the gut‐brain axis, the purposeful alteration of the gut microbiome through diet or probiotics may be an effective therapeutic target to improve AD outcomes.^75^ Gut inflammation, escalated intestinal leakiness, and dysbiosis of the gut microbiome can be associated with aging‐related illnesses, such as AD. When enterococcus strains from the guts of healthy infant mice were administered to aging mice, intestinal inflammation, leaky gut, dysbiosis, and motor dysfunction were ameliorated. The probiotics altered the gut microbiome such that tight junctions were reinforced, attenuating gut leakiness and inflammation. Additionally, probiotics also spurred the formation of tight junctions by bolstering bile salt hydrolase performance, leading to an elevation of taurine in the gut.^52^ Moreover, rats were subject to intracerebroventricular injection of β‐amyloid with half of this group receiving probiotics. Compared to the non‐probiotic administered group, the probiotic‐treated group exhibited improved navigation in the Morris water maze. This group also displayed improved long‐term potentiation and enhanced antioxidant/oxidant biomarker balance.^76^ Furthermore, after exercise and administration of probiotics, APP/PS1TG mice demonstrated substantial cognitive and motor improvement, as observed in the Morris Maze Test. Notably, the amount of beta‐amyloid plaques in the hippocampus was reduced. An increase in B.thetaiotaomicron and L.johnsonii bacteria in the gut microbiome could be associated with the cognitive amelioration in these mice.^77^ Moreover, altering the gut microbiome through probiotic treatment shows significant therapeutic promise in alleviating gut dysbiosis and inflammation in neurological disorders such as AD.

A wide range of probiotics has been implemented as potential therapies for gut dysbiosis in experimental models of AD. Administration of Morinda Officinalis‐derived fructooligosaccharides (OMO) into AD‐rodent models demonstrated ameliorative effects on AD symptoms. As a potential prebiotic, OMO was given to D‐galactose‐ and Aβ1‐42‐induced deficient rats and behavioral experiments indicated significant improvement in both rat's memory and learning abilities. Upon sequencing, OMO supported diverse and stable microbe populations in the gut. Furthermore, histological alterations exhibited a decrease in neuroinflammation, downregulation of AD‐biomarkers, Tau and Aβ1‐42, and amelioration of neuronal apoptosis.^78^ In addition, probiotics, such as Lactobacilli and Bifidobacteria, may effectively attenuate the drastic metabolic permutations caused by insulin resistance in AD. Insulin resistance can spur the development of AD by inciting changes in serum levels of insulin and fasting blood sugar, as well as altering the lipid profile. When AD experimental models were given Lactobacilli and Bifidobacteria as probiotics, the glycemic condition associated with AD was more efficiently regulated.^79^

Endogenous and microbial metabolites heavily influence surrounding microbial populations and consequently, cognitive function. Specific metabolites produced from gut microbiota processes have been associated with AD‐linked cognitive dysfunction. Succinic acid, DOPAC, and mannitol demonstrate a substantial correlation with AD‐induced cognitive impairment. Another metabolite, D‐proline, can be associated with a reduction in amyloid P in AD‐afflicted cerebrospinal fluid. Therapeutically, amyloids may be eliminated in the brain by capitalizing on d‐proline producing bacteria, which in turn, would attenuate AD‐induced cognitive dysfunction.^80^ Furthermore, modulation of endogenous metabolites via chemical agents may provide advantageous AD treatment options. Xanthoceraside's (XAN) therapeutic effects on treating AD were measured via behavioral testing and H&E staining observation. Sequencing fecal samples revealed reversal of AD‐inducing gut dysbiosis due to the shift of bacterial population ratios such as the Firmicutes/Bacteroidetes. Metabolomics study indicated that XAN is also able to modulate endogenous metabolites, heavily influencing microbe populations and consequently improving cognition.^81^ Notably, Bile acid (BA) production and metabolism, regulated by the liver and gut microbiota, becomes defective during AD progression. When compared to normal participants, AD subjects demonstrated substantially lower levels of primary BA and significantly higher amounts of secondary BA. Upregulations of deoxycholic acid, along with its glycine and taurine altered conformations were observed, indicating gut microbiota induced 7α‐dehydroxylation of cholic acid, which is correlated with cognitive deterioration.^82^

The gut‐brain axis is a preeminent phenomenon and provides an abundance of implications throughout the body bolstering its potential as a therapeutic mechanism. Targeted intervention of the gut‐brain axis may give rise to favorable outcomes in AD and other neurodegenerative disorders. Gut microbiota influence many neurophysiological processes, such as maintenance of BBB integrity, neural development, aging, and CNS immune activation.^83^ Accumulating research indicates that modulation of microbial composition through prebiotics, probiotics, and metabolites, may reverse AD‐linked dysbiosis, thereby providing improvements in cognition and functional outcomes. Investigating the underlying mechanisms behind the promising findings may provide valuable insight into creating novel therapies for AD. Further elucidation is imperative in establishing optimal dosage, timing, and pre/probiotic agents.

## 4 POTENTIAL TRANSLATION OF AD TREATMENT TO STROKE BY TARGETING GUT MICROBIOME

Knowledge of pathological similarities between stroke and AD may facilitate the diagnosis and treatment of dementia in stroke. Both are present with inflammation‐induced cerebral atrophy and secondary neuronal cell death, consequently exacerbating cognitive impairments and physiological damage.^33^, ^34^, ^35^, ^36^, ^37^, ^38^, ^39^, ^40^, ^41^, ^64^, ^84^ Additionally, the role of microglia in both stroke and AD may hint at overlapping pathological mechanisms.^40^, ^41^, ^85^ The risk of stroke‐induced dementia may be determined by the presence of prior ischemic injury, vascular comorbidities, WML’s, and genetic predisposition.^28^ Therefore, a deeper understanding of the underlying mechanisms behind AD will allow for more effective treatment. An approach targeting the gut microbiome for diagnosis and treatment of stroke‐induced dementia shows therapeutic promise. Like AD, stroke can spur gut leakiness and dysbiosis, diminishing beneficial microbes and elevating opportunistic bacteria in the gut.^78^ In addition, white matter injury, associated with stroke and AD cognitive decline, can be linked to gut dysbiosis with a reduction of gut‐microbial diversity.^67^, ^80^ Furthermore, altering the gut microbiome may be therapeutic against stroke and AD cognitive impairment, as the gut microbiome can play a neuroprotective role.^83^

Despite the pathological similarities between stroke and AD,^64^ such as cognitive decline and dementia, the mechanisms behind neuronal death in both diseases are distinctly different and comparable. Stroke occurs when cerebral blood flow to the brain is interrupted, depriving neuronal cells of oxygen and nutrients. Cell death in stroke involves various mechanisms, including apoptosis and necrosis. Necrosis occurs within the first few minutes of damage when sudden decrease in blood flow leads to significant decrease in ATP, an essential molecule to maintain functioning Na^+^/K^+^ pumps in neurons.^86^ The pumps fail to regulate ion concentrations with insufficient ATP, allowing sodium ions to accumulate within the neuron. Cellular edema develops, and the cell membrane ruptures, resulting in nuclei degradation. Additionally, accumulation of calcium ions may lead to mitochondrial dysfunction during ischemic stroke. Damaged mitochondria release cytochrome C to activate caspase and initiate cell death.^87^ The activated caspase may lead to either apoptosis or autophagy depending on the amount of energy in the cell.^86^, ^88^ Post‐stroke cell death promotes inflammatory activity that causes secondary cell death, further damaging the brain. Unlike stroke, AD‐induced cell death relies on amyloid β (Aβ) plaques.^84^ Aβ is often observed in senile plaques and is synthesized from amyloid precursor protein (APP) by cleavage of enzymes.^89^ When APP is metabolized by β‐ and γ‐secretase in the plasma membrane, Aβ is released outside of the cell or broken down in lysozymes. Studies hypothesize that changes in Aβ level promote cascades responsible for cell death. Additionally, Aβ activates pro‐inflammatory cytokines, such as TNF‐α and PIKA, in astrocytes and microglia.^90^ These pro‐inflammatory mechanisms promote apoptosis and neurodegenerative symptoms. Due to the significant difference in the mechanisms of neuronal death, careful consideration is necessary when extending diagnoses and treatments between diseases. Despite similar cognitive symptoms and the profound effects of gut microbiota in AD, careful evaluations must be considered before applying similar microbiota therapy from AD to stroke due to the mechanistic differences in the etiologies of these two diseases. Accordingly, utmost caution is necessary when assessing the converging cognition‐relevant brain region (eg, hippocampus) affected in both diseases that likely promotes the overlapping symptomatologic dementia. Similarly, careful consideration must be exercised when contemplating microbiome‐based treatments that will be possibly require disease‐tailoring such therapy to stroke distinct from AD, and vice versa.

Cognitive deficits and dementia symptoms are present in both AD and stroke due to similarities in affected brain regions, despite the difference in origin of the diseases. Studies suggest that the hippocampus and neocortex are involved in early stages of AD where synaptic loss and neurodegeneration first occur,^91^ correlating to cognitive dysfunction involving memory. Symptoms of AD‐induced dementia is associated with cerebral choline acetyltransferase (CAT) and acetylcholinesterase (AChE) activity, which indicates Aβ levels and cognitive impairment.^40^, ^92^ Patients with severe AD were observed to have an average of 25% to 33% decrease in cortical AChE activity. Additionally, mild and moderate severities of AD were found to have 30% AChE activity in the cerebral cortex compared to the control groups.^92^, ^93^ Based on the findings, AD‐induced cognitive deficits and dementia arise from structural damage in the hippocampus, neocortex, and cerebral cortex. Although the cellular mechanism of neuronal death and structural damage differs from AD, stroke‐induced dementia and cognitive impairments similar to AD arise in similar brain areas, specifically hippocampus and cerebral cortex.^94^ Dementia post‐stroke can also occur when other brain structures, such as the thalamus and frontal lobe, are damaged either through secondary cell death via inflammation or primary lesions, which results in observable AD‐like symptoms involving memory and cognition.

Alterations of gut microbiota demonstrated lower levels of neuroinflammatory activity in AD, and similar anti‐inflammatory mechanisms may be present for stroke‐induced inflammation in the same brain region, alleviating symptomatologic dementia similar to AD, due to the similar pathogenesis of AD and stroke in regards to gut microbiome (Table 1). Microbiota is evidently associated with inflammatory activity in the brain due to the communication between the gut and brain. Physiological changes in the brain induce pro‐inflammatory immune T‐cells to circulate from the GI tract into the brain towards the damage site,^42^, ^73^ promoting inflammatory activity and exacerbating neuronal cell death. Treatments targeting therapeutic alterations to the gut microbiome has not only restored healthy gut functions and alleviated gut inflammation, but also slowed AD cognitive decline.^52^, ^77^, ^95^ Additionally, cognitive dysfunction in AD was seen to be associated with metabolites produced by gut microbiota, including Succinic acid, DOPAC, and mannitol.^80^ Furthermore, probiotic treatments that alter microbiota diversity decrease levels of Aβ plaque, which lowers inflammatory activity and pro‐inflammatory cytokines.^68^, ^77^, ^90^, ^96^ Clearly, microbiota alterations possess therapeutic mechanisms that reduce AD outcomes. Through careful examination, AD therapy targeting gut microbiota may also be effective in alleviating post‐stroke dementia and AD‐like cognitive symptoms. Post‐stroke dementia may benefit from gut microbiome therapy used in AD due to evidence of relationship between stroke and gut microbiome via gut‐brain axis,^44^, ^45^, ^46^ correlation between post‐stroke inflammation and microbiota,^42^, ^43^ and converging brain regions involved in both diseases that cause similar dementia and cognitive symptoms.^91^, ^92^, ^94^ However, due to the clear difference in the pathology of cell death and the onset of both diseases, studies are clearly warranted to test whether AD gut treatment can be extended to post‐stroke dementia therapy. Notwithstanding, we are not claiming that gut‐microbial dysfunction is the primary causation of AD or stroke. Rather, we believe microbial dysfunction is the peripheral consequence of the neurological diseases due to the gut‐brain axis, which may worsen existing disease symptoms by promoting further neuroinflammatory activity. Therefore, restoring gut deficit may improve disease outcomes in the brain.

**TABLE 1:** A summary of the mile‐stone discoveries linking cognitive decline in stroke and AD pathology. Gut dysbiosis can be associated with both stroke and AD cognitive impairment and, therefore, may be an effective therapeutic target for treatment of these diseases

Studies	Discovery
Yin et al. (2015)	There is a correlation between stroke and gut dysbiosis. The gut microbiome of stroke and transient ischemic attack patients contained increased levels of opportunistic pathogens and decreased beneficial genera.^48^ These patients also exhibited reduced levels of trimethylamine‐N‐oxide (TMAO), a promoter of atherogenesis that is produced by gut microbiota. Decreased TMAO levels may be a mechanism of stroke induction.
Chen et al. (2016)	White matter hyperintensities can be associated with cognitive impairment in stroke and dementia. In aged post‐stroke patients, cognitive decline can be linked to astrocyte damage and dysfunction of gliovascular activity with the BBB.^39^ Clasmatdendrosis may also be a factor inducing white matter hyperintensities, further exacerbating post‐stroke cognitive injury and dementia.
Crapser et al. (2016)	Infection after stroke is a major factor causing stroke‐induced mortality, and risk of infection increases with age. In both young and aged MCAO mice, stroke spurred gut leakiness and bacterial translocation from the gut to surrounding organs. However, the young mice overcame the infection, while the aged mice endured worsened hypothermia, weight loss, and immune impairment, indicating sepsis.^50^
Singh et al. (2016)	Stroke lesions cause gut microbiota alterations, consequently influencing stroke outcomes through immune mechanisms. Reduced diversity and augmented levels of bacteroidetes are common after stroke. Recolonization of germ‐free mice with post‐stroke microbiota increases lesion volume and functional deficits, and also upregulates T‐cell polarization in the intestinal immune compartment and injured brain.^45^ Fecal transplantation of healthy microbiota improves stroke outcome and ameliorates brain‐lesion induced dysbiosis.
Xu et al. (2016)	AD‐induced cognitive decline can be associated with metabolites produced by gut microorganisms. In AD, cognitive deterioration has been significantly correlated with Succinic acid, DOPAC, and mannitol. Since D‐proline may diminish amyloid P in cerebrospinal fluid, utilizing d‐proline producing bacteria as a therapeutic implement in AD may be effective.^80^
Vogt et al. (2017)	Through the examination of fecal samples from AD patients with dementia and participants without AD‐related cognitive impairment, AD’s effect on gut microbiota diversity was revealed.^73^ The gut microbiome became much less varied and developed lower levels of Firmicutes and Bifidobacterium as well as a high amount of Bacteroidetes.
Zhang et al. (2017)	Microbiota composition and SCFA levels differ in AD mice compared to wild‐type. SCFA fluctuation influences metabolic pathways and consequently exacerbates amyloid deposition and cognitive deficits.^68^
Singh et al. (2018)	Microbiome composition modulates stroke outcomes. Germ‐free mice were compared to recolonized Ex‐GF and SPF mice and recolonization reduces stroke volumes and increased cytokine and microglia/macrophage amounts. Microbiome induced neuroprotection was not observed in lymphocyte deficient mice, indicating that lymphocytes play a role in microbiome‐mediated neuroprotection.^2^
Spychala et al. (2018)	When young mice underwent MCAO, their gut microbiome mirrored the gut flora of healthy elder mice. Further modifying the microbiome of young mice to match the aged mice exacerbated stroke symptoms, escalated mortality rates, and elevated inflammatory cytokine levels.^51^ On the other hand, changing the microbiome of stroke‐afflicted aged mice to reflect a young mouse ameliorated symptoms and increased viability.
Wendeln et al. (2018)	Peripheral immune stimulation, training and tolerance, modulates pathology of neurological diseases. In an AD mouse model, immune training furthers cerebral β‐amyloidosis, and tolerance reduces it. Immune stimulation also alters post‐stroke pathology.^96^
Abraham et al. (2019)	Pathogenesis of AD can be slowed exercise and probiotic treatment via the gut microbiome. APP/PS1TG mice were exercised and administered probiotics. Improvements were seen in the Morris Maze Test due to augmented B. thetaiotaomicron levels and decreased levels of beta‐amyloid plaques in the hippocampus via L. johnsonii.^77^
Ahmadi et al. (2020)	The administration of enterococcus strains from healthy infant mice to aging mice as a probiotic alleviated gut inflammation, endothelial leakiness, and motor impairment. The probiotics improved inflammation and gut leakiness by fortifying tight junctions through the elevation of bile salt hydrolase activity.^52^

Targeting the gut microbiome may represent one of many potent therapeutic modalities for AD and stroke. Gut microbiome may help alleviate neuroinflammation, improve cognitive functions or retard neurodegeneration and cognitive deficits inherent in both AD and stroke. Treatment strategies incorporating microbiota treatment, such as prebiotics and probiotics, have demonstrated promising results for AD by reducing hippocampal Aβ plaques and improving cognitive performance in mouse models^56^, ^77^, ^78^, ^81^, ^82^; the same may potentially translate over to stroke. Probiotic treatments, such as *Lactobacillus Plantarum* ZDY2013, *Clostridium butyricum*, or probiotic mixtures, and bacterial metabolites were seen to improve stroke‐related disorders.^97^, ^98^ However, these supplementary treatments were not beneficial in more severe stroke cases. Complete repopulation of the GI tract via fecal microbiota transplantation may be most suitable in severe stroke‐induced deficits.^51^

## 5 CONCLUSION

The pathogenesis of stroke parallels AD in terms of cognitive impairment, dementia, and its associated brain pathology. Both demonstrate secondary neuronal cell death due to uncontrolled inflammatory response, causing structural damage to the brain and leading to cognitive deficits. In stroke, pro‐inflammatory activity induces cerebral atrophy, which is similar to the neurodegenerative mechanisms seen in AD. With AD being the more prevalent type of dementia, AD research demonstrating a correlation between microbiome populations and cognitive impairments may be similarly extended to stroke‐induced dementia. Additionally, gut microbiome regulation may be utilized as a possible therapeutic strategy to treat both AD and stroke. Due to the correlation between microbiome populations and CNS diseases, altering gut microbiome populations through diet modulation or probiotics may improve cognitive functions. Gut microbiomes that closely approximate AD‐like cognitive impairment may manifest as the same gut microbiota altered in stroke, and targeting specific pro‐inflammatory microbiota may improve cognitive symptoms in CNS diseases. However, due to the disease‐specific cell death mechanisms in stroke and AD, microbiota alterations in AD treatment may not translate effectively over to stroke treatment, and vice versa. Careful and rigorous investigations are required to evaluate the applicability of microbiome‐based treatment to each disease. Additionally, gut microbiota is neither the key factor in stroke and AD pathology nor the only treatment target for CNS diseases. Gut dysbiosis is the consequence of CNS diseases possibly due to the gut‐brain axis that worsens cognitive impairment,^99^, ^100^, ^101^ and it is one of many potent treatment targets that may regulate neuroinflammation and reduce stroke outcomes. Elucidating the key role of gut microbiomes in CNS disease pathology may elucidate new potential targeted candidates for stroke therapy. Recognizing the gut as a major source of inflammation in stroke pathology warrants integration of the gut in addition to the brain in pre‐existing stroke tools for diagnosis and treatment. In particular, distinct gut microbiomes may precipitate stroke dementia, resembling AD cognitive decline, and suggests a cross‐disease exploration of their pathologies and therapies.

## CONFLICT OF INTEREST

The authors declare no conflict of interest.
